# Patient’s and Practionner’s Experiences of a First Face-to-Face vs. Remote Orthodontic Consultation: A Randomized Controlled Trial

**DOI:** 10.3390/healthcare11060882

**Published:** 2023-03-17

**Authors:** Carole Charavet, Fiona Rouanet, Sophie Myriam Dridi

**Affiliations:** 1 Département d’Orthodontie, Faculté de Chirurgie Dentaire, Université Côte d’Azur, 06300 Nice, France; 2 Centre Hospitalier Universitaire de Nice, Institut de Médecine Bucco-Dentaire, Unité d’Orthodontie, 06300 Nice, France; 3 Laboratoire MICORALIS UPR 7354, Université Côte d’Azur, 06000 Nice, France; 4 Département de Parodontologie, Faculté de Chirurgie Dentaire, Université Côte d’Azur, 06300 Nice, France; 5 Centre Hospitalier Universitaire de Nice, Institut de Médecine Bucco-Dentaire, Unité de Parodontologie, 06300 Nice, France

**Keywords:** teleorthodontics, remote consultation, teleconsultation, teledentistry, satisfaction, video-assisted remote consultation

## Abstract

(1) Aim: The purpose of this study was to assess patients’ and practitioners’ reported experience measures (PREMs) following a first standard orthodontic consultation (face-to-face consultation) versus a first orthodontic teleconsultation (video-assisted remote orthodontic consultation).; (2) Materials and Methods: This study was designed as a randomized controlled trial in which 60 patients were randomly allocated to two groups. In the control group, patients received a first face-to-face consultation (*n* = 30) whereas, in the test group, patients received a first orthodontic teleconsultation (*n* = 30). Patients as well as the orthodontic practitioners completed questionnaires after the experience. (3) Results: From the patients’ point of view, overall satisfaction was comparable between the control group and the test group (*p* = 0.23). Quality of communication with the clinician, understanding of the explanations provided and a sense of privacy were also comparable between the two groups. However, from the practitioners’ perspective, overall satisfaction after the face-to-face consultation was significantly higher than after the first remote consultation (*p* < 0.01). (4) Conclusions: In the context of a first orthodontic consultation, teleorthodontics appears to be an interesting and complementary approach to a classical face-to-face appointment, but which should by no means become systematic.

## 1. Introduction

Since the 20th century, the use of new technologies has increased exponentially and the development of telemedicine [1,2,3,4,5,6,7,8,9] is naturally part of this trend. Remote management of orthodontic treatment has also been introduced and is known as “teleorthodontics ” [10]. The sudden onset of the COVID-19 pandemic has boosted remote medical care [11,12,13,14,15,16,17,18,19,20,21,22,23], including orthodontics. In a study published by Saccomanno et al. [24], teleorthodontics are shown to be a valuable tool for orthodontic care follow-up, in the context of an emergency as well as in normal circumstances, to save time and money without unduly reducing quality of care. More recently, in 2022, Saccomanno et al. [25] conducted a systematic review on the utility of teleorthodontics for orthodontic emergencies in a context of the COVID-19 sanitary crisis. Out of the 1695 articles available on the four databases selected (PubMed, Science Direct, Cochrane and SciELO), eight articles were included and analyzed. The authors concluded that teleorthodontics is a relevant contribution to the management of orthodontic emergencies in case of contact person limitation. Recently, Lamb et al. [26] investigated the adaptations of orthodontic practice during the COVID-19 pandemic and the adaptations which were expected to stay after the crisis by a survey of 34 questions, sent by email in June 2021 to 1000 orthodontist specialists. With a hundred and sixty surveys returned from 38 different states across the United States (16% response rate), the results showed that during the COVID-19 crisis, the utilization of teleorthodontics increased from 8% to 68%. However, only 45% of these orthodontists responded that they would continue to use teleorthodontics after the pandemic. After the pandemic, teleorthodontics were expected to be employed for new patients, such as for aligners treatment monitoring, but not for fixed treatment follow-up.

Furthermore, teleorthodontics could be a precious service for patients with difficulties in planning an in-office appointment. France is currently facing a lack of medical resources in certain areas of the country, known as “medical deserts”, hindering access to medical facilities, including orthodontic care. A cross-sectional questionnaire study carried out by Mathivanan et al. [27] investigated the interest of teledentistry for rural dental practice among general practitioners in and around Coimbatore district, Tamil Nadu, India. The survey was distributed to 200 dentists and 73 of them responded. Of the 37% of the dentists who have responded to the questionnaire, the results showed that 73% of the dentists estimated that teledentistry could offer access of specialists to the rural population. Additionally, 96% of the respondents agreed that teledentistry would be the future of rural dental practice. In the same vein, Berndt et al. [28] investigated the feasibility of a general practitioner carrying out interceptive orthodontic treatment under the control of an orthodontist by teledentistry for disadvantaged children (referral to an orthodontist is not possible). They included 30 children treated by a general practitioner under the control of an orthodontist by teledentistry and 96 children treated by orthodontic residents. The improvement of peer assessment rating (PAR) index was found in the two groups with 35.6% for the teledentistry group and 44.1% for the control group, leading to no difference between the two groups (*p* < 0.001). Therefore, teleorthodontics could greatly benefit patients who have difficulty reaching a practice or a hospital.

Rouanet et al. [9] explored also in a recent systematic review the relevance of teleorthodontics tool, in which an extensive panel of study designs was included to investigate this topic exhaustively. In total, 22 studies were included and analyzed, with a very variable levels of evidence. The studies were mainly from Europe but also from other continents (America, Oceania, Asia), which indicates that teleorthodontics is already used, or at least studied, throughout the world. These studies are mostly recent, with the majority being published between 2019 and 2021. According to the results of their systematic review, they summarized the advantages and disadvantages of the teleorthodontics. The advantages include: the resolution of certain orthodontic emergencies; anticipation for the next appointment (especially in case of an emergency); follow-up in the context of a pandemic, without risk of contamination; better follow-up of retention appointments; better compliance and oral hygiene with remote communication; easy communication between patients and practitioners; reliability of tools and ease of use; attractiveness of the practice, modernity; solution to be explored to facilitate access to orthodontics in medical deserts; time-saving as a result of fewer chairside appointments (less travel to the office) and shorter appointments on average (overall total duration); and saving money (travel costs). On the other hand, disadvantages are as follows: danger of fully remote treatment (to be avoided); not all phases of an orthodontic treatment can be carried out remotely; question of confidentiality, security, data protection; possible impoverishment of the human relationship and, therefore, of the quality of the therapeutic alliance; dissatisfaction and/or difficulty of use (patients less at ease with the new technology or find difficulty in the acquisition of intra-oral photos/scans); investment for the practitioner, such as computer equipment, subscriptions to follow-up services, cost of a scan box, etc., and similarly for the patient in some cases; lack of interest/fear; no shortening of treatment time; and a lack of evidence/data investigating all aspects of telemedicine in orthodontics. Moreover, in a recent systematic review and meta-analysis, Alam et al. [29] explored the future of orthodontics. Out of 634 publications from four databases (PubMed-MEDLINE, Web of sciences, Cochrane and Scopus), the authors included 17 articles based on the inclusion and exclusion criteria at the end of process of the study selection. Four categories were highlighted as emerging technologies: 3D printing, computer-aided design and computer-aided manufacturing (CAD/CAM), biopolymers and teleorthodontics. They also mentioned in their discussion section that the COVID-19 pandemic has also amplified the importance of the topic of teleorthodontics. Finally, according to another literature review conducted by Maspero et al. [30], teleorthodontics will thus play a role in the near future.

Several recent studies have already investigated the reliability of teleorthodontics and demonstrated encouraging results both in terms of effectiveness [31,32,33,34] and satisfaction. Hansa et al. [31] compared the total treatment time, number of appointments, number of refinements, time taken up to the first refinement, number of emergency appointments and accuracy of predicted tooth positions between clear aligner treatment assisted by dental monitoring (*n* = 45 patients) versus clear aligner treatment without any system of teleorthodontics (*n* = 45 patients). The number of appointments were reduced by 33,1% in the dental monitoring group compared to the control group. Furthermore, a significant reduction in the time of the first refinement was demonstrated in the dental monitoring group, which also indicated a better aligner tracking in the dental monitoring group. Byrne et al. [32] explored the level of satisfaction among 59 patients and 62 clinicians after video consultations during the COVID-19 pandemic. They found that 76% of the patients considered a remote consultation to be more convenient than a face-to-face consultation and 66% stated that they would like more remote consultation appointments in the future. Additionally, 90% of the clinicians considered that a remote consultation was appropriate. These results agree with studies conducted by Parker et al. [33,34] who also found very encouraging results from both patients’ and clinicians’ points of view.

However, to the best of our knowledge, no study has measured patient and clinician satisfaction following an initial remote orthodontic consultation. The objective of the present randomized controlled trial was therefore to investigate patients’ and practitioners’ reported experience measures (PREMs) following a first standard face-to-face consultation versus a first orthodontic teleconsultation (video-assisted remote orthodontic consultation).

## 2. Materials and Methods

Registration: This randomized controlled trial (RCT) was approved by the Department of Clinical Research and Innovation of Nice (file number: 447). The trial was registered on the ClinicalTrials.gov website (NCT 05646277). All patients were verbally informed of the purposes and monitoring of the study, and they all signed an informed consent form.

**Figure 1:**
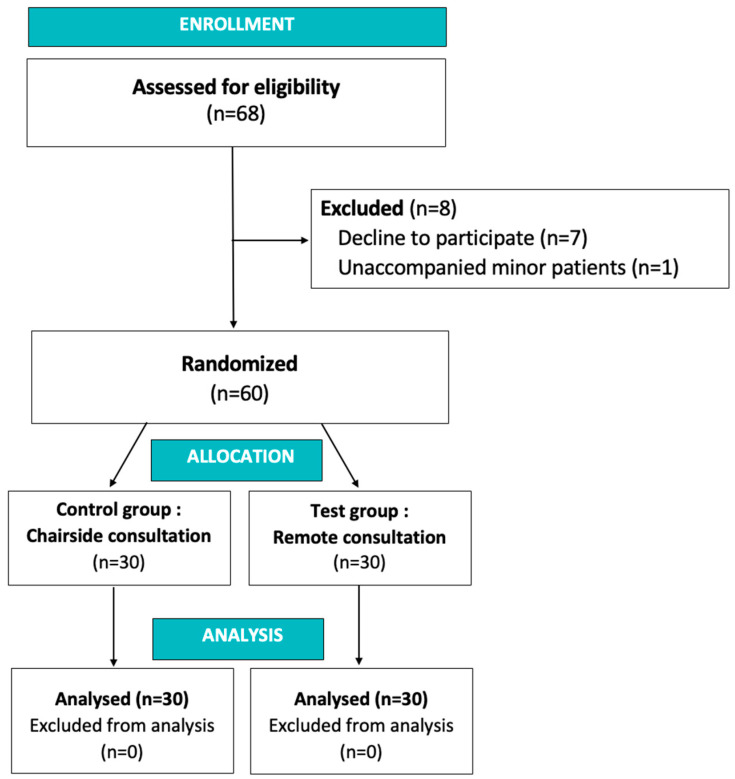
CONSORT Flow diagram.

Study design: The study was designed as a single center, two-arm, parallel-group, randomized controlled trial (RCT) with a 1:1 allocation ratio and evaluated patients’ and practitioners’ reported experience (PRE) following a first classic chairside consultation versus a first orthodontic teleconsultation (orthodontic remote consultation). Sixty consecutive patients requesting a first orthodontic consultation in the orthodontic unit of Nice University Hospital were included and randomly assigned to the control group (classic chairside consultation; *n* = 30) or to the test group (teleconsultation; *n* = 30). A CONSORT flow diagram is illustrated in Figure 1. Only the patients who requested a first orthodontic consultation could be included in the trial. Children not accompanied by their legal guardians (under 18 years old) were excluded. The practitioners involved in this study attended three calibration meetings, in which the objectives of the trial, the protocols and the assessment method were jointly reviewed and agreed upon. There were no changes to the protocol after trial initiation.

Procedure:

- Control group (CG): Patients had an initial face-to-face chairside consultation under classic conditions. After the experience, patients completed a questionnaire. Note tipper: the term “initial consultation” refers to the first orthodontic consultation, where no dental cast or dental X-ray are performed;
- Test group (TG): Patients received a first orthodontic teleconsultation via video communication using a computer equipped with a webcam, a microphone and a speaker. After the experience, the patients completed a questionnaire;
- Practitioners: After each consultation, the two orthodontic practitioners completed a questionnaire.

Data collection:

- Patient characteristics: The following parameters were collected at baseline for each patient: gender, age and knowledge of telemedicine (yes/no). Distance, time and cost of travel to the hospital were also recorded;
- Outcome data: Patients’ and practitioners’ reported experience were assessed using questionnaires containing open and closed questions as well as a 0–10 visual analog scale (VAS) after the experience. Practitioners provided each patient with a comprehensive explanation of the use of the VAS and how to enter the outcome measure, according to Wewers and Lowe [35], and collected the questionnaires.
  ⚬ For both groups: patients were asked to rate their overall satisfaction, the quality of communication with the clinician, their understanding of the explanations provided and their sense of confidentiality;
  ⚬ For the test group only: an additional questionnaire was given in which patients were asked whether they would recommend the procedure to a friend, whether they would undergo the teleconsultation again and whether they would repeat the experience for a follow-up orthodontic consultation. If the answer was negative, the patient was asked to mention the reason. Finally, patients rated their satisfaction with the technical aspects of the consultation;
  ⚬ For the practitioners: a questionnaire was also delivered after each consultation. Overall satisfaction, quality of the communication with the patient, feasibility of establishing a clear and definite clinical diagnosis and deciding the need for a complete orthodontic examination (photos, radiographs, dental casts) were evaluated. Additionally, after the teleconsultation, practitioners were asked whether they would recommend the method to a colleague and if they were willing to repeat this type of consultation.

Finally, the duration of the consultation was also recorded in the two groups as well as the technical issues that arose during the teleconsultation in the test group.

Statistical Analysis: Data were presented as mean ± standard deviation (SD). Groups were compared using Student’s t test. Results were considered significant at the 5% critical level (*p* < 0.05)

## 3. Results

Patient Characteristics: Sixty patients (33 female; 27 male) aged 16.2 ± 10.5 years were included in the study and randomly allocated to the test group (teleconsultation, *n* = 30) or the control group (classic chairside consultation, *n* = 30). Patient characteristics of each group are shown in Table 1.

One out of two patients had never heard of the concept of telemedicine. The average distance from the patient’s home to the hospital was 9.4 ± 8.7 km for a travel duration of 24.9 ± 13.2 min and a cost of 3.4 ± 3.8 EUR, with no difference between the two groups (Table 2).

**Figure 2:**
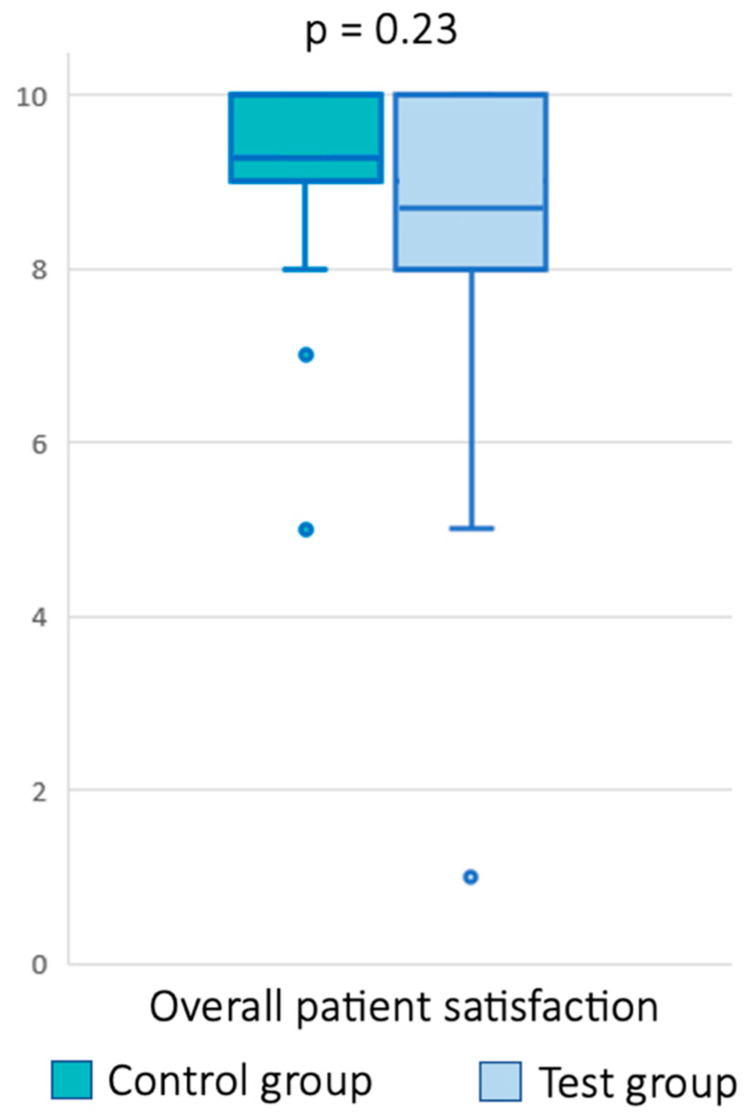
Overall patient satisfaction after a face-to-face consultation (control group) and after a teleconsultation (test group). The dots represent the outlier values.

Outcomes in both groups: The response rate was 100% among patients. Overall satisfaction was comparable between the control group with a classic face-to-face consultation (9.21 ± 1.3) and the test group with a teleconsultation (8.69 ± 1.9) (*p* = 0.23) (Figure 2). The quality of the communication with the clinician was rated 9.28 ± 1.2 in the control group compared to 9.16 ± 1.5 in the test group. The difference between the two groups was not statistically significant (*p* = 0.74). Additionally, in each group, one out of 30 patients reported that they did not fully understand the explanations, and there was thus no difference between the two groups. Finally, 96.7% of the patients in the control group considered the face-to-face consultation to be confidential. All patients in the test group considered that the video consultation was confidential.

Outcomes in the test group: 10.3% of the patients in the test group would not recommend a remote consultation to a friend. Furthermore, 17.2% of patients would not wish to repeat this first orthodontic teleconsultation, nor would they wish to repeat the experience in a follow-up orthodontic consultation. Among them, 100% considered that face-to-face contact with the practitioner is essential during a consultation. Finally, patient satisfaction with the use of the technology was rated 8.4 ± 2.2.

**Figure 3:**
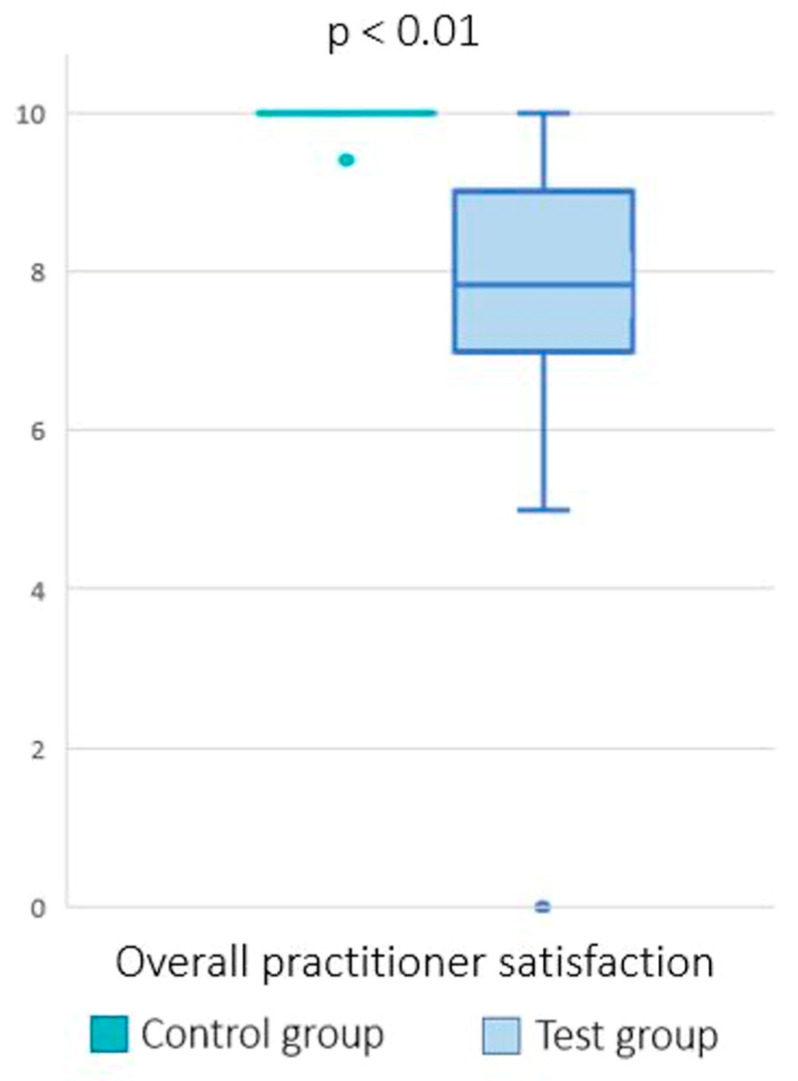
Overall practitioner satisfaction after a face-to-face consultation (control group) and after a teleconsultation (test group). The dots represent the outlier values.

Outcomes from the practitioners: The response rate was 100% among clinicians. The overall satisfaction was 9.8 ± 0.2 after the consultation of the control group compared to 7.65 ± 1.9 after the consultation of the test group. The difference between the two groups was statistically significant (*p*< 0.01) (Figure 3).

The quality of the communication with the patient was 9.83 ± 0.4 after the consultation among the control group compared to 8.1 ± 1.8 after the consultation among the test group. The difference between the two groups was statistically significant (*p* < 0.01). While a clear and definite clinical diagnosis could be established during all face-to face consultations, this was not possible for 53.3% of cases during the remote consultation. However, in 87.5% of cases, the latter procedure was still sufficient to determine whether the patient needed additional investigations (X-rays/dental casts/photos).

Duration of the consultation: The average duration of the teleconsultation was 12.14 ± 3.1 min compared to 14.47 ± 1.9 min for the face-to-face consultation. The difference between the two groups was significant (*p* < 0.01).

Technical issues (test group): For 23.3% of the teleconsultations, a technical problem arose. In 42.8% of the cases, very short, inconsequential, sound interruptions occurred, except for one consultation which had to be reinitiated (the sound was completely cut off). In 28.7% of cases, there was a very short, minor interruption of the internet connection. Another issue was inadequate lighting, hindering intraoral examination. In such cases, the patient was asked to move or to use a sun shield.

## 4. Discussion

This randomized controlled trial was the first, to our knowledge, to compare patients’ and practitioners’ experience following an initial remote orthodontic consultation versus a first traditional face-to-face orthodontic consultation. The two groups were homogeneous for all baseline characteristics, except for age, which was significantly higher in the control group. Following a first orthodontic teleconsultation, patient satisfaction was high, while results were more mixed from the practitioners’ perspective.

Overall patient satisfaction in the test group was relatively high and, although slightly lower than among patients in the control group, the difference was not statistically significant. Similar results were reported in the medical literature. In a randomized controlled trial, Buvik et al. [36] investigated patients’ reported outcomes following a remote orthopedic consultation by telemedicine (199 patients) compared to a standard face-to-face consultation (190 patients). In that study, 99% of the patients evaluated the remote consultation as very satisfactory or satisfactory, and 86% said that they would prefer a video-assisted consultation for their next consultation. In the field of dentistry, Parker et al. [33] evaluated 111 satisfaction questionnaires from patients who attended a video consultation and found that one-third preferred the video consultation to a standard consultation, while one-third were neutral. In addition, 90% of the patients would recommend the video consultation. On the other hand, in the present study, patients in the control group remained extremely satisfied with their consultation despite the relatively long travel time and distance as well as the time slots that required them to miss work or school. In the study conducted by Buvik et al. [36], 99% of patients in the control group were also very satisfied or satisfied after their face-to-face consultation. In terms of quality of communication with the clinician, understanding of the explanations and their sense of confidentiality during the experience, satisfaction was comparable between the two groups, in agreement with results reported by Parker et al. [33]. Indeed, they asked their patients if they could easily talk about the care they received and 72.2% and 20.7% of patients “strongly agreed” and “agreed”, respectively. Additionally, whereas in the Parker et al. [33] study, patients who reported a preference for face-to-face consultations mentioned poor internet connection as one of the negative reasons, in the present RCT, only one disadvantage was noted, namely the lack of direct contact with the practitioner, despite some technical issues raised by practitioners during 23.3% of the video consultations. In fact, patient satisfaction with the technology was relatively high. It is worth noting that a similar percentage of technical problems was found in the Byrne et al. [32] study (27%), where most problems were related to connection issues (sound loss and temporary video interruption). All in all, although the standard face-to-face consultation is still quite appropriate, the remote consultation in orthodontics in the context of a first consultation is a tool that patients greatly appreciate and consider satisfactory or very satisfactory.

The average travel time from patients’ home to the hospital was 24.9 ± 13.2 min, to which must be added the equivalent duration of the return journey, as well as the time spent in the waiting room. Saccomanno et al. [24] had already reached an estimate of 50 min (28 min for the round trip, 7 min for parking and 15 min in the waiting room) for the average time spent by patients in addition to the actual appointment. Thus, a remote consultation saves time. It should also be noted that in this RCT, the time spent on the consultation itself was significantly shorter by about two and a half minutes during the video consultation compared to the standard consultation. This reduction in consultation time during video appointments had already been reported by Saccomanno et al. [24], and also mentioned by Byrne et al. [32], although the duration of the virtual consultation was not investigated in the latter study.

However, from a practitioner’s point of view, the results are less encouraging, even though, in most cases, the video consultation was sufficient for the practitioner to know whether or not the patient needed a complete orthodontic examination, which is the initial goal of a first consultation. Orthodontic practice systematically requires obtaining dental casts and X-rays to make a complete and thorough diagnosis and initiate treatment.

Moreover, although a clinical examination seems relatively easy to perform remotely, functional and periodontal assessment remains a more complex procedure. A classic face-to-face appointment is therefore essential before starting orthodontic treatment.

However, an initial remote orthodontic consultation is still useful in many situations. For example, those living far from a medical facility can thus have access to a practitioner, allowing useful and early detection of orthodontic disorders (complex and/or urgent cases). In addition, during public health crises such as the COVID-19 epidemic, this tool remains the only available link with a practitioner. It can also reassure parents and/or adult patients and raise their awareness of the importance of orthodontic treatment.

A first remote consultation is thus a communication tool and should by no means be considered as a substitute for conventional diagnosis or treatment. Recently, Rouanet et al. [9] investigated the relevance of teleorthodontics tools in a systematic review and also concluded that “ teleorthodontics is an interesting and complementary tool that is, in no way, a systematic alternative to face-to-face orthodontic appointments in the office”.

Finally, some limitations should be addressed. This present RCT is a single-center study that would benefit from being expanded to other centers. Due to the lack of published material on the subject in the field of orthodontics, the sample power calculation was based on our active patient records. The cost of the journey home–hospital was 3.4 ± 3.8 EUR for both groups, which is quite low and could therefore influence the results and make it less attractive to carry out a remote consultation. In addition, it would have been interesting to carry out each remote consultation twice independently by the two orthodontist practitioners to check whether their results were consistent. Further, this study is based solely on satisfaction questionnaires and, considering the rather encouraging results, teleorthodontics should now be more widely investigated (including the data protection, informed consent of patient, long-term results, etc.).

## 5. Conclusions and Perspective

Given the limitations of this first randomized controlled trial on this topic, the following conclusions could be stated:

- The overall satisfaction of patients who received a first remote orthodontic consultation was high and not significantly different from patients who received a first standard face-to-face orthodontic consultation, including in terms of quality of communication with the practitioner, understanding of explanations and their sense of confidentiality;
- However, overall clinician satisfaction was significantly lower after the teleconsultation compared to the traditional consultation.

Teleorthodontics, in the context of a first consultation, appears to be an interesting alternative and complementary approach to a classical face-to-face appointment, but which should by no means become systematic. A first remote consultation is thus a communication tool and should by no means be considered as a substitute for conventional diagnosis or treatment.

Finally, in many countries, such as France, the distribution of health professionals is not homogeneous, leading to “medical deserts”. Could telemedicine help to open up territories?

## Figures and Tables

**Table 1:** Patient baseline characteristics by group (*n* = 60). The values given are average ± standard deviation or number (%).

	Control Group (*n* = 30)	Test Group (*n* = 30)	Comparison *p*-Value
Gender			0.84
Female	16 (53.3)	17 (56.7)	
Male	14 (46.7)	13 (43.3)	
Age (years)	19.6 ± 12.9	12.9 ± 5.8	0.01 *

* *p* < 0.05.

**Table 2:** Comparison of patients’ knowledge of telemedicine and access to hospital according to group (*n* = 60). The values given are average ± standard deviation or number (%).

	Control Group (*n* = 30)	Test Group (*n* = 30)	Comparison *p*-Value
Knowledge of telemedicine			1
Yes	15 (50)	15 (50)	
No	15 (50)	15 (50)	
Patient’s home to hospital journey			
Distance (km)	9.5 ± 3.5	9.3 ± 6.4	0.86
Duration (minutes)	26.5 ± 14.9	23.3 ± 10.6	0.32
Cost (EUR)	3.3 ± 2.8	3.6 ± 1.4	0.56
